# P-2167. "Clinical Utility of Cell-free Next-generation Sequencing, Single Center Experience"

**DOI:** 10.1093/ofid/ofae631.2321

**Published:** 2025-01-29

**Authors:** Osama Ibrahim, Ralph Rogers, Dimitrios Farmakiotis

**Affiliations:** Brown University, Boston, Massachusetts; Brown University, Boston, Massachusetts; Brown University, Boston, Massachusetts

## Abstract

**Background:**

Timely diagnosis of infectious diseases is crucial, but it can be challenging. The literature about impact of Karius in clinical care is still insufficient. Overall, Karius has been very effective on selected patients but the clinical impact is difficult to assess due to absence of gold standard comparator. In addition, a negative result doesn’t always mean a “true negative” but means that the organism’s DNA wasn’t detected. Also, Identification of non-relevant results has been reported.

**Figure:**
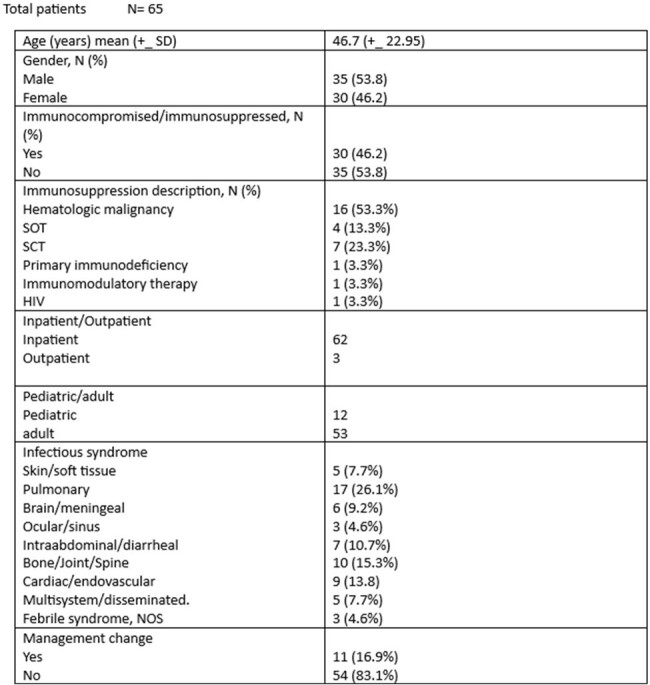

**Methods:**

This is a retrospective cohort study of patients who received a Karius testing between January 2018 until March 2023 at Lifespan institution in Rhode Island. Clinical and demographic data was abstracted from the electronic medical record. Quality checks were applied to the data to remove duplicates and errors. Descriptive statistics were reported using Microsoft excel. Clinical impact was abritrated after review of each case by three independent investigators. We predefined clinical impact categories criteria based on criteria from previous studies. Each case was reviewed by each investigator independently and disparities were solved via consensus and discussions. inter-rater reliability was assessed using Cohen's Kappa coefficient.

**Figure:**
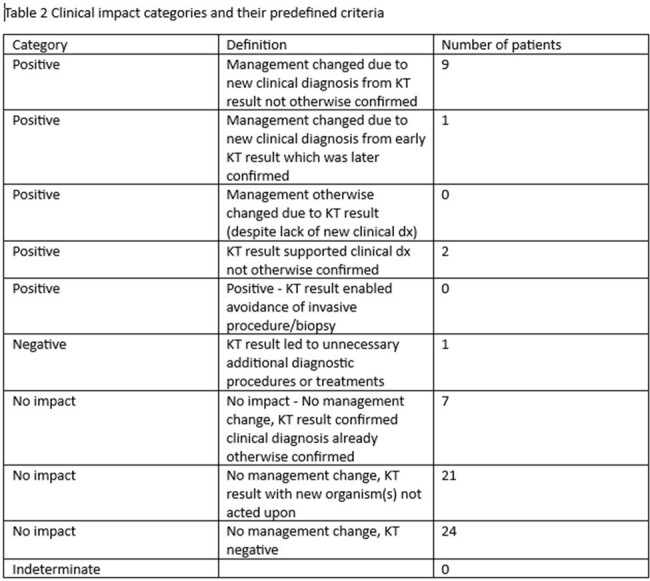

**Results:**

Overall, 65 patient had Karius testing and included 35 male and 30 female. 30 patients were immunocompromised and 16 of them had hematologic malignancy. Mean age was 46.7 and the study included 12 pediatric and 53 adult patient. The most common infectious syndrome was Pneumonia/pulmonary followed by osteoarticular and endovascular. Management was changed in 11/65 patients. The Karius testing had positive impact in 12 patients, negative impact in 1 patient and no impact in 52 patients. Positive percent agreement with conventional testing was 0.49 and negative percent agreement was 0.83

**Figure:**
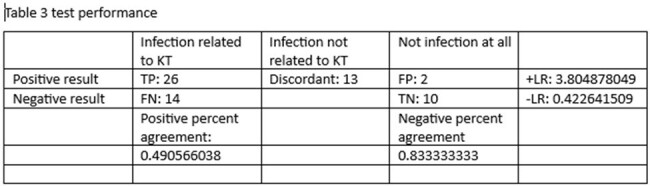

**Conclusion:**

Cell-free next-generation sequencing has a valuable role in diagnosis and management of infections in challenging cases especially in patients with high pretest probability. Further research is needed to define the population who would benefit most from it.

**Disclosures:**

All Authors: No reported disclosures

